# ANS‐SCMC: A matrix completion method based on adaptive neighbourhood similarity and sparse constraints for predicting microbe‐disease associations

**DOI:** 10.1111/jcmm.70071

**Published:** 2024-09-19

**Authors:** Haoran Wen, Xue Zhong, Lieqing Lin, Langcheng Chen

**Affiliations:** ^1^ School of International Education Guangdong University of Technology Guangzhou Guangdong China; ^2^ School of Computer Science Guangdong University of Technology Guangzhou Guangdong China; ^3^ Center of Campus Network and Modern Educational Technology Guangdong University of Technology Guangzhou Guangdong China

**Keywords:** adaptive neighbourhood similarity, iterative gradient descent, microbe‐disease associations, sparse constraint‐based matrix completion

## Abstract

The use of matrix completion methods to predict the association between microbes and diseases can effectively improve treatment efficiency. However, the similarity measures used in the existing methods are often influenced by various factors such as neighbourhood size, choice of similarity metric, or multiple parameters for similarity fusion, making it challenging. Additionally, matrix completion is currently limited by the sparsity of the initial association matrix, which restricts its predictive performance. To address these problems, we propose a matrix completion method based on adaptive neighbourhood similarity and sparse constraints (ANS‐SCMC) for predict microbe‐disease potential associations. Adaptive neighbourhood similarity learning dynamically uses the decomposition results as effective information for the next learning iteration by simultaneously performing local manifold structure learning and decomposition. This approach effectively preserves fine local structure information and avoids the influence of weight parameters directly involved in similarity measurement. Additionally, the sparse constraint‐based matrix completion approach can better handle the sparsity challenge in the association matrix. Finally, the algorithm we proposed has achieved significantly higher predictive performance in the validation compared to several commonly used prediction methods proposed to date. Furthermore, in the case study, the prediction algorithm achieved an accuracy of up to 80% for the top 10 microbes associated with type 1 diabetes and 100% for Crohn's disease respectively.

## 1 INTRODUCTION

Microbes are the major root cause of many human diseases.^1^ Therefore, the study of the relationship between microbes and various diseases is of great significance for clinical treatment, human health care and drug development.^2^ The current microbe‐related diseases are generally infected first, then treated and prevented in the end.^3^ If the microbe–disease associations can be predicted by machine learning models for disease prevention and treatment prognosis development, it has extensive research and application value. With the accumulation of biomedical data and the synergistic development of computational technology, the application of machine learning models to predict the correlation between microbes and diseases has been widely studied and applied.^4^ Currently, modelling algorithms used in this field mainly include network‐based methods, deep learning‐based methods and matrix‐based methods.^5^

Network based methods typically infer possible correlations by analysing the network topology structures constructed in multiple databases. This approach relies on the structural information between different networks and identify association patterns.^6^ For example, Zou et al.^7^ proposed a method based on a double random walk (RW) on heterogeneous networks. Yin et al.^8^ proposed a label propagation (LP) method based on multi‐similarity fusion. Although LP and RW algorithms are efficient and easy to use, but their prediction methods cover limited biological information.^9^ Li et al.^10^ proposed the KATZBNRA model based on the binary network recommendation algorithm and KATZ. Wang et al.^11^ proposed a network embedding (NE) method based on heterogeneous networks and global graph feature learning. Among them, the KATZ Measure method can simultaneously reconstruct potential associations with large‐scale networks, but its calculation is based on the GIP kernel similarity may have a certain impact on the known associations.^12^ On the other hand, the concept of meta‐paths used in NE can clearly capture basic higher‐order proximity. However, as network information increases, the complexity of training embeddings also increase.^13^

The rate of the usage of deep learning technologies is increasing in the field of bioinformatics including graph convolutional network (GCN),^14^ network distance analysis,^15^ deep capsule networks,^16^ graph attention mechanisms (GAT),^17^ fully convolutional networks (FCN),^18^ deep sparse autoencoder neural networks (SAE)^19^ and other new methods.^20^, ^21^, ^22^, ^23^ These technologies provide new research approaches for microbe–disease association prediction by building complex nonlinear models and mining deep features and relationships in data. Many scholars have also applied deep learning technologies to microbe–disease association prediction. For example, Lu et al. proposed a method based on autoencoders and GCN to predict the association between microbes and diseases.^24^ Liu D et al. proposed a method based on graph attention networks (GAT) to predict the association between microbes and diseases.^25^ Wang et al. proposed a method for predicting potential microbe–disease associations based on multi‐source features and deep learning, which used deep sparse autoencoder neural networks (SAE).^14^ GCN improves translation invariance on non‐matrix structured data, but its flexibility and scalability need to be improved.^26^ GAT has a significant effect on the aggregation of graph neural networks, but it has some difficulties in the aggregation of high‐order neighbourhoods and is sensitive to parameter initialization.^27^ In the prediction of microbe–disease associations, SAE can effectively mine important features, compress secondary features, and generate lower‐dimensional and sparser abstract features, but it cannot clearly distinguish between the activity and hiddenness of nodes, and the selection of sparsity parameters is also difficult.^28^

Matrix based methods, such as matrix factorization and matrix completion, mainly work by decomposing the original high‐dimensional input matrix into two lower dimensional matrices. These two smaller matrices are continuously updated through optimization algorithms during the iteration process, with the aim of making their product as close as the original matrix as possible. For example, Liu et al.^29^ proposed a non‐negative matrix factorization (NMF) based on graph regularization. Yang et al.^30^ proposed a multi‐similarity bilinear matrix factorization method based on similarity constrained matrix factorization (SC‐MF). Xu et al.^31^ proposed a new collaborative weighted non‐negative matrix factorization method based on collaborative matrix factorization (collaborative‐MF). The method based on matrix decomposition is a method that can discover deeper potential connections. Meanwhile, the spatial complexity of matrix decomposition is relatively low. However, the MF‐based method has more parameters. Therefore, the parameter selection is more difficult and the model training time is longer.^32^ Another matrix‐based method is matrix completion. The matrix completion method restores the matrix of missing values to a complete matrix by decomposing a matrix of missing values into two or more matrices mainly through matrix decomposition, and then multiplying these decomposed matrices to obtain an approximate matrix of the original matrix. For example, Shi et al. proposed a binary matrix complementation method.^33^ Long et al.^34^ proposed a graph regularized non‐negative matrix complementation method. The matrix decomposition‐based complementation model is a method that avoids complex matrix singular value decomposition, and it can be implemented in a distributed environment, but it belongs to a kind of non‐convex optimization, which may result in a non‐globally optimal solution.^35^ The matrix‐based methods mentioned above rely on the similarity information between microbes and diseases for prediction. Moreover, the sparsity issue of the initial association matrix also significantly affects the predictive performance of such methods.

Considering the limitations of the existing matrix completion methods in terms of the reliability of similarity information and the sparsity of the association matrix, we propose a matrix completion method based on adaptive neighbourhood similarity and sparse constraints (ANS‐SCMC). We first calculated the similarity between the matrices of disease Gaussian interaction contour kernels and microbe Gaussian interaction contour kernels in the correlation matrix. Based on the obtained disease similarity and microbial similarity information, we use WKNKN to preprocess the microbe–disease feature representation. Next, we obtain the adaptive neighbourhood similarity information of microbes and diseases based on Laplacian flow pattern local learning method. Then, based on adaptive neighbourhood similarity information, we used the matrix completion with sparse constraints to start with the existing matrix information, decompose the preprocessed loss function into low rank sub‐matrixes, and continuously capture the correlations of the original matrix through gradient descent, continuously filling in the missing parts of the original correlation matrix. Finally, we obtained the final predicted score matrix. To validate the effectiveness of ANS‐SCMC, we compared its predictive performance with six other state‐of‐the‐art MDA identification models. The results demonstrated that ANS‐SCMC outperformed the other models in terms of prediction accuracy. In the case study, ANS‐SCMC has been demonstrated as a reliable and effective model with strong predictive capability for microbe–disease association.

## 2 MATERIALS AND METHODS

### 2.1 Data preparation

We obtained the relevant data from the human microbe–disease association database, which is available from the Human Microbe–Disease Association Database (HMDAD, http://www.cuilab.cn/hmdad).These data are mainly derived from microbe studies based on 16 s RNA sequencing, which only give information at the genus level. The database includes 483 experimentally validated human microbe–disease associations involving 39 different human diseases and 292 microbes. We have compiled 450 different associations based on different evidence. In our study, these microbe–disease associations were constructed into an adjacency matrix A. If there is an association between a certain microbe $m\left(i\right)$ and a certain disease $d\left(j\right)$, the corresponding value of $A\left(m\left(i\right),d\left(j\right)\right)$ is 1, and if there is no association, the corresponding value of $A\left(m\left(i\right),d\left(j\right)\right)$ is 0. In addition, we defined two variables $n_{m}$ and $n_{d}$, which is used to represent the number of microbe species and the number of disease species involved in the study.

### 2.2 Kernel similarity of Gaussian interaction profiles of microbes

Under the assumption that microbes with functional similarity are usually associated with similar diseases and therefore share similar interaction patterns with diseases, we adopted a method to compute microbe similarity from known microbe–disease association networks using Gaussian interaction profiles of microbe to kernel similarity. This process typically consists of two steps: firstly, we defined binary vectors $\mathrm{AM}\left(m\left(i\right)\right)$ declaring the interaction profiles of microbe $m\left(i\right)$, which are used to record whether the microbe $m\left(i\right)$ are associated with each disease; Then, we calculated the kernel similarity between each pair of microbe base on the Gaussian interaction profiles of the microbes. After calculating the similarity between pairs of microbes, we can construct a Gaussian interaction distribution kernel similarity matrix MF as show in Equation (1).

(1) $$\mathrm{MF}\left(m\left(i\right),m\left(j\right)\right)=\exp\left(-\theta_{m}{\left\|\left.\mathrm{AM}\left(m\left(i\right)\right)-\mathrm{AM}\left(m\left(j\right)\right)\right\|\right.}^{2}\right)$$

(2) $$\theta_{m}=\theta_{m}^{\prime }/\left[\frac{1}{n_{m}}\sum_{i=1}^{n_{m}}{\left\|\mathrm{AM}\left(m\left(i\right)\right)\right\|}^{2}\right]$$

Here, $\theta_{m}$ regulates the normalized kernel bandwidth based on the new bandwidth parameter $\theta_{m}^{\prime }$ as in Equation (2). Each $\mathrm{MF}\left(m\left(i\right),m\left(j\right)\right)$ record represents the Gaussian interaction profile kernel similarity between the microbe $m\left(i\right)$ and $m\left(j\right)$.

### 2.3 Gaussian interactive contour kernel similarity for disease

As described in Section 2.2, it is assumed that functionally similar microbes are usually associated with similar diseases. Therefore, we can calculate the Gaussian interaction profile kernel similarity of diseases in a similar way to microbes. We define binary vector $\mathrm{AD}\left(m\left(i\right)\right)$ to represent the interaction spectrum of the disease $d\left(i\right)$, which records whether each disease $d\left(i\right)$ is associated with each microbe. Then, we calculate the kernel similarity between each pair of diseases based on the Gaussian interaction profile of the disease. After calculating the similarity between disease pairs, we can construct a Gaussian interaction distribution kernel similarity matrix DF, as shown in Equation (3):

(3) $$\mathrm{DF}\left(d\left(i\right),d\left(j\right)\right)=\exp\left(-\theta_{d}{\left\|\mathrm{AD}\left(d\left(i\right)\right)-\mathrm{AD}\left(d\left(j\right)\right)\right\|}^{2}\right)$$

(4) $$\theta_{d}=\frac{\theta_{d}^{\prime }}{\frac{1}{n_{d}}\sum_{i=1}^{n_{d}}{\left\|\mathrm{AD}\left(d\left(i\right)\right)\right\|}^{2}}$$

where the kernel bandwidth parameter $\theta_{d}^{\prime }$ is calculated by normalizing a new bandwidth parameter $\theta_{d}^{\prime }$ with the average number of associations between each disease and the microbe, as shown in Equation (4).

### 2.4 Methodology overview

**FIGURE 1:**
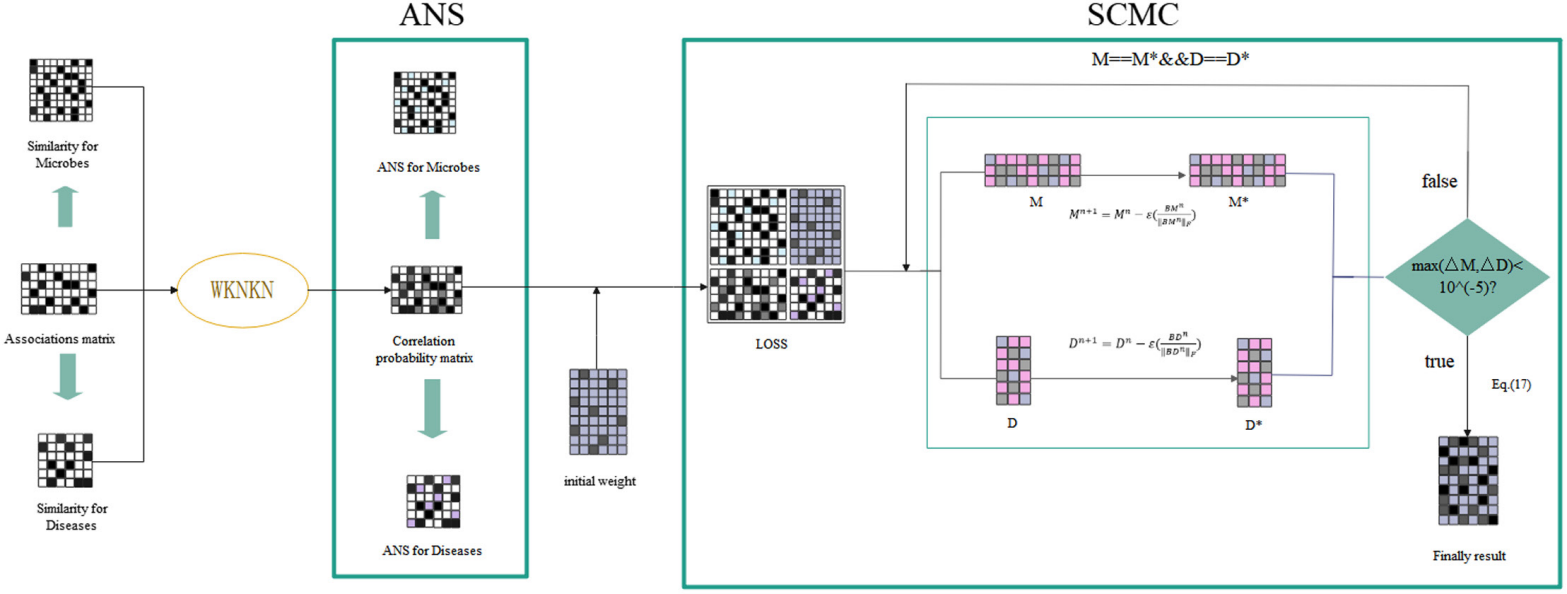
Flowchart of ANS‐SCMC.

In this study, ANS‐SCMC was utilized to predict possible relationships between microbes and diseases. We first downloaded human microbe–disease associations, calculated the Gaussian interaction contour kernel similarity between diseases and microbes in them, and used WKNKN to reduce the sparsity of the disease‐microbe association matrix. Then the adaptive domain similarity matrix was obtained based on the disease‐microbe association matrix. Finally, we used the matrix completion with sparse constraints to update the resulting loss function with cyclic gradient descent to obtain the final result. Figure 1 depicts the algorithmic flow of ANS‐SCMC, which consists of three parts: the Correlation probability matrix with WKNKN algorithm, Adaptive neighbourhood similarity (ANS) and Matrix Completion with Sparse Constraints (SCMC).

### 2.5 Correlation probability matrix with WKNKN algorithm

To characterize the relationship between microbes and diseases, we used the WKNKN algorithm to reduce the sparsity of the disease‐microbe association matrix A. An experimentally proven microbe (disease) is associated with at least one disease (microbe), but there are still undiscovered potential interactions in the disease‐microbe association matrix A. Therefore, we propose to use WKNKN as a preprocessing step to explore the possibility of potential interactions between microbes and diseases.

Step 1: For each microbe $m_{i}$, we use the Gaussian interaction profile similarity matrix MF obtained in 2.2 to find k known microbes adjacent to it, and use the scores of these microbes to infer the interaction possibility spectrum of $m_{i}$. We formulate WKNKN as shown in Equation (5):

$$A_{m}\left(m_{i},:\right)=\frac{1}{Z_{m}}\sum_{n_{m}=1}^{k}w_{n_{m}}A_{m}\left(m_{n_{m}},:\right)$$

$$w_{n_{m}}=\alpha^{n_{m}-1}\mathrm{MF}\left(m_{i},m_{n_{m}}\right)$$

(5) $$s.t.\alpha \leq 1，Z_{m}=\sum_{n_{m}=1}^{k}\mathrm{MF}\left(m_{i},m_{n_{m}}\right)$$

where $l_{1}$ to $l_{k}$ are the k nearest known neighbours of $m_{i}$ in descending order, $w_{n_{m}}$ is the weight coefficient.

Step 2: Similarly, for each disease $d_{j}$, we also infer the probability spectrum of its interaction based on the Gaussian interaction profile similarity matrix DF obtained in 2.3, as shown in Equation (6):

$$A_{d}\left(:,d_{j}\right)=\frac{1}{Z_{d}}\sum_{n_{d}=1}^{K}w_{n_{d}}A_{d}\left(:,d_{n_{d}}\right)$$

$$w_{n_{d}}=\alpha^{n_{d}-1}\mathrm{DF}\left(d_{n_{d}},d_{j}\right)$$

(6) $$s.t.\leq 1,Z_{d}=\sum_{n_{d}=1}^{K}\mathrm{DF}\left(d_{n_{d}},d_{j}\right)$$

where $d_{1}$ to $d_{K}$ are the K nearest known neighbours represented by $d_{j}$ in descending order, $w_{n_{d}}$ is the weight coefficient.

Step 3: Finally, we denote the obtained average of $A_{m}$ and $A_{d}$ as Q and fill the blank entries in matrix A with the corresponding values in Q as shown in Equation (7) and (8):

(7) $$Q=\left(A_{m}+A_{d}\right)/2$$

(8) $$A_{i,j}=\left\{\begin{array}{c} Q_{i,j},A_{i,j}=0\\ A_{i,j},A_{i,j}\neq 0 \end{array}\right.$$

We call this matrix processed by the WKNKN algorithm the filled correlation matrix and name it $A_{W}$.

### 2.6 Adaptive neighbourhood similarity

The traditional Laplacian flow learning method mainly reconstructs the local structural characteristics of the data manifold by constructing an adjacency matrix, ultimately achieving the purpose of enhancing the smoothness of data in its linear and non‐linear space. This is based on an assumption: if two data points are adjacent in the geometric structure of the original space, then they should also maintain similarity in the new representation space. However, when constructing such a graph, traditional methods do not fully consider the actual number of data sets, which may result in the inclusion of many unnecessary interactions in the graph, thus failing to accurately reflect the internal geometric structure of the data. In order to overcome the limitation that the Laplacian graph may only produce trivial solutions, we adopt a method that does not rely on distance, see Equation (9):

$$\min_{w}\;\sum_{i<j}^{n_{m}}\;\left(∥a_{i}-a_{j}{∥}_{2}^{2}w_{\mathrm{ij}}+r_{0}w_{\mathrm{ij}}^{2}\right)$$

(9) $$s.t.\;0\leq w_{\mathrm{ij}}\leq 1$$

where $a_{i}$ and $a_{j}$ represent the i and j lines of A, $w_{\mathrm{ij}}$ represents the similarity between microbe i and microbe j, $n_{m}$ is the known number of row vectors in the association matrix, and the regularization term ${r_{0}w}_{\mathrm{ij}}^{2}$ ensures that all optimal solutions are close to the data point $x_{i}$ and have the same probability $\frac{1}{n_{m}}$. Here, the regularization parameter $r_{0}$ is used to adjust the control ability of the adaptive neighbourhood size in the local structure similarity graph matrix. This parameter can be understood as a priori knowledge that helps us determine the range of domain assignment.

Optimization of the solution:

The above equation its equivalent to Equation (10):

(10) $$\min_{W，L}\;\;\mathrm{tr}\left(A^{T}\mathrm{LA}\right)+\frac{r_{1}}{2}∥W{∥}_{F}^{2}+\frac{r_{2}}{2}∥L-D_{W}+W{∥}_{F}^{2}$$

where L is the matrix of temporary proxy variables used to approximate $\left(W-D_{W}\right)$, $D_{W}$ is the diagonal matrix of W, $r_{1}$ and $r_{2}$ are regular term hyperparameter. So W and L are solved as in Equation (11) and (12):

(11) $$W\leftarrow \arg\;\mathrm{mi}n_{W}\frac{r_{1}}{2}∥W{∥}_{F}^{2}+\frac{r_{2}}{2}∥L-D_{W}+W{∥}_{F}^{2}$$

(12) $$L\leftarrow \arg\;\mathrm{mi}n_{L}\mathrm{tr}\left(A^{T}\mathrm{LA}\right)+\frac{r_{2}}{2}∥L-D_{W}+W{∥}_{F}^{2}$$

Based on this we can obtain Equation (13), (14):

(13) $$W\leftarrow {\left(r_{1}E+r_{2}E\right)}^{-1}\left(r_{2}D_{W}-r_{2}L\right)$$

(14) $$L\leftarrow {\left(r_{2}E\right)}^{-1}\left(r_{2}D_{W}-r_{2}W-AA^{T}\right)$$

where E is the unit matrix of $n_{m}\times n_{m}$.

The iterative updating stops when the condition $\max\left(∥W^{t}-W^{t-1}{∥}_{F}^{2}+∥L^{t}-L^{t-1}{∥}_{F}^{2}\right)<10^{-3}$ is satisfied, for this we will obtain $W^{t}$ as the final similarity matrix MS for the microbes. Similarly, we can obtain the similarity matrix *DS* for the diseases based on the same method.

**FIGURE 2:**
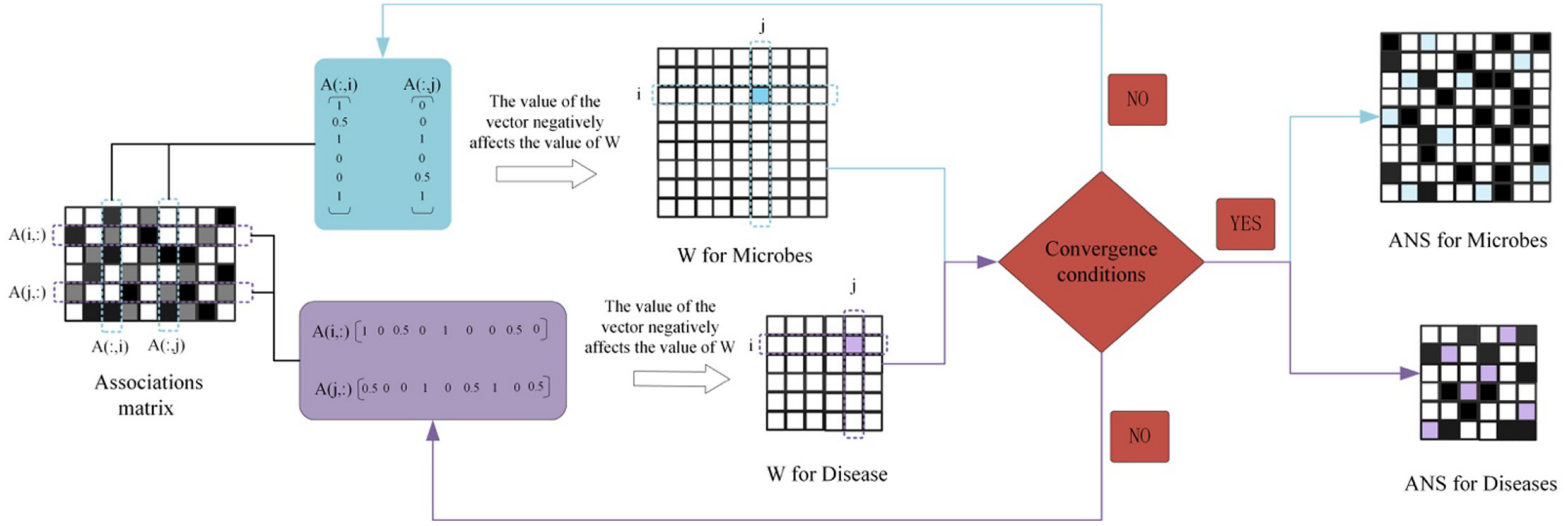
The details of the steps for adaptive neighbourhood similarity.

In the calculation of the adaptive similarity matrix, we allocate weights between adaptive and optimal neighbours by dynamically calculating the differences in vector data for each column or row of the known correlation matrix, thereby obtaining the similarity matrix of microbe or disease data. Our model can perform local manifold structure learning on the information of the known correlation matrix, adaptively balancing the differences in known correlation information and the minimization of prior information. During the process of learning the data similarity matrix, we continuously adjust the value of weight W to preserve the adaptive local structure of the data. The details of the adaptive similarity steps are shown in Figure 2.

### 2.7 Matrix completion with sparse constraints

SCMC is a matrix completion method. Its core idea is to approximate the target matrix by iteratively generating an approximate matrix through the inner product of two sub‐matrices. Specifically, for a microbe–disease association matrix $A\in R^{n_{m}*n_{d}}$ containing missing information, where the number of microbes and the number of diseases is represented by $n_{m}$ and $n_{d}$, respectively. We decompose the preprocessed matrix into two‐dimensional sub‐matrices: the microbe feature matrix $M\in R^{n_{m}*r}$ and the disease feature matrix $D\in R^{n_{d}*r}$, $\left(M,D\sim N\left(0,\frac{1}{\surd r}\right)\right)$, and simulate $A_{W}$ by calculating the inner product of these two matrices, where r represents the number of low‐dimensional space. In this process, the disease‐microbe association information is mapped to a common low‐dimensional matrix, and we use this method to predict the possibility of disease‐microbe association. In the process of matrix completion, we continuously narrow the gap between the approximate matrix and the target matrix through gradient descent to optimize the above sub‐matrices. In order to prevent overfitting of the model, SCMC calculates a complexity sub‐matrix that contains a penalty term to simulate the difference between the simulated models. In addition, SCMC retains the original information of the disease‐microbe association and continuously adds the predicted association information to the sub‐matrix model to continuously adjust and update the sub‐matrix. Therefore, for a specific microbe $m_{i}$ and disease $d_{j}$, we express its association probability as Equation (15):

(15) $$p_{i,j}=p\left(A_{i,j}^{W}=1|m_{i},d_{i}\right)=\frac{1}{\left(1+\exp{\left(m_{i}d_{j}^{T}\right)}^{-1}\right)}$$

or in matrix form, as in Equation (16).

(16) $$p\left(A_{W}|M,D\right)=\prod_{i,j}{\left(p_{i,j}^{A_{i,j}^{W}}{\left(1-p_{i,j}\right)}^{1-A_{i,j}^{W}}\right)}^{w_{i,j}}$$

Here, $A_{i,j}^{W}$ represents the elements in row i and column j of $A_{W}$, $p_{i,j}$ represents the association probability between microbe $m_{i}$ and disease $d_{j}$, while $m_{i}$ and $d_{j}$ represent the $i^{\mathrm{th}}$ row and $j^{\mathrm{th}}$ row of the microbe submatrix M and disease submatrix D, $p_{i,j}^{A_{i,j}^{W}}$ is a conditional probability expression that represents the probability of an association between microbe $m_{i}$ and disease $d_{j}$, given a given microbe $m_{i}$ and disease $d_{j}$. In addition, we added the initial weights $w_{i,j}$ of microbe $m_{i}$ and disease $d_{j}$ to improve the model's fitting ability and performance. We derived $p\left(A_{W}|M,D\right)$ through Bayesian reasoning, as shown in Equation (17):

(17) $$p\left(M,D|A_{W}\right)\propto p\left(A_{W}|M,D\right)p\left(M\right)p\left(D\right)$$

The loss function is obtained as in Equation (18).

(18) $$\text{LOSS}=\sum_{i,j}w_{i,j}\left\{\ln\left(1+\exp\left(m_{i}d_{j}^{T}\right)\right)-A_{i,j}^{W}m_{i}d_{j}^{T}\right\}+\lambda_{R}\left({\left\|M\right\|}_{2}^{2}+{\left\|D\right\|}_{2}^{2}\;\right)$$

To improve the accuracy of the prediction method, we introduced a sparse constraint coefficient $\lambda_{R}$ to adjust the sparsity of M and D. In addition, considering that similar microbes are likely to be associated with similar diseases, we further expanded the loss function. Specifically, we used the microbe similarity matrix $\mathrm{MF}\in R^{n_{m}*n_{m}}$, where each entry ${\mathrm{MF}}_{i,j}$ represents the similarity between microbes $m_{i}$ and $m_{j}$ Similarly, we used the disease similarity matrix $\mathrm{DF}\in R^{n_{d}*n_{d}}$, where each entry ${\mathrm{DF}}_{i,j}$ represents the similarity between diseases $d_{i}$ and $d_{j}$. We explained the association between similar diseases and similar microbes by reducing the distance between microbe characteristics, as shown in formula (19):

(19) $$\sigma \left(M^{T}\left(M_{\mathrm{MF}}-\mathrm{MF}\right)M\right)=\frac{1}{2}\sum_{i=1}^{n_{m}}\sum_{j=1}^{n_{m}}\mathrm{MF}\left(i,j\right){\left\|M\left(i,:\right)-M(j,:)\right\|}_{2}^{2}$$

Similarly, the similarity between diseases is minimized as in Equation (20):

(20) $$\sigma \left(D^{T}\left(D_{\mathrm{DF}}-\mathrm{DF}\right)D\right)=\frac{1}{2}\sum_{i=1}^{n_{d}}\sum_{j=1}^{n_{d}}\mathrm{DF}\left(i,j\right){\left\|D\left(i,:\right)-D(j,:)\right\|}_{2}^{2}$$

We introduce regularization term 2 and regularization term 3 into 1 and add two additional adjustable parameters $\lambda_{\alpha }$ and $\lambda_{\beta }$. Finally, we transform the loss function into Equation (21):

(21) $$\text{LOSS}=\sum_{i,j}w_{i,j}\left\{\ln\left(1+\exp\left(m_{i}d_{j}^{T}\right)\right)-A_{i,j}^{W}m_{i}d_{j}^{T}\right\}+\lambda_{R}\left({\left\|M\right\|}_{2}^{2}+{\left\|D\right\|}_{2}^{2}\right)+\lambda_{\alpha }\sigma \left(M^{T}\left(M_{\mathrm{MF}}-\mathrm{MF}\right)M\right)+\lambda_{\beta }\sigma \left(D^{T}\left(D_{\mathrm{DF}}-\mathrm{DF}\right)D\right)$$

SCMC uses the iterative gradient descent method (AdaGrad) to optimize the model. During the iteration process, we write the partial derivative of the loss function as formula (22) and (23) to guide the optimization:

(22) $$\mathrm{BM}=\frac{\partial \text{LOSS}}{\partial M}=\left\{W⊙\left[P-A_{W}\right]\right\}D+2\lambda_{R}M+2\lambda_{\alpha }\left(D_{\mathrm{MF}}-\mathrm{MF}\right)M$$

(23) $$\mathrm{BD}=\frac{\partial \text{LOSS}}{\partial D}=\left\{W^{T}⊙\left[P-A_{W}^{T}\right]\right\}M+2\lambda_{R}D+2\lambda_{\beta }\left(d_{\mathrm{DF}}-\mathrm{DF}\right)D$$

where BM represents the partial derivative of LOSS over M, BD represents the partial derivative of LOSS over D, ⊙ denotes the Hadamard product, and the submatrices M and D will be updated according to a specific formula. For details, please refer to Equation (24) and (25):

(24) $$M^{n+1}=M^{n}-\varepsilon \left(\frac{{\mathrm{BM}}^{n}}{{\left\|{\mathrm{BM}}^{n}\right\|}_{F}}\right)$$

(25) $$D^{n+1}=D^{n}-\varepsilon \left(\frac{{\mathrm{BD}}^{n}}{{\left\|{\mathrm{BD}}^{n}\right\|}_{F}}\right)$$

(26) $$∆M={\left\|M^{n+1}\right\|}_{F}-{\left\|M^{n}\right\|}_{F}$$

(27) $$∆D={\left\|D^{n+1}\right\|}_{F}-{\left\|D^{n}\right\|}_{F}$$

Among them, ε represents the learning rate, which is a key parameter in the iterative optimization process. Based on experience, we usually set ε to a fixed value 0.1 to simplify the calculation. The superscript n represents the current number of iterations. The update of the submatrices M and D will continue until the end condition $\max\left(∆M,∆D\right)<10^{-5}$ is reached; ${\left\|\mathrm{BM}\right\|}_{F}$ and ${\left\|\mathrm{BD}\right\|}_{F}$ represent the Frobenius normal form of BM and BD respectively, as shown in Equation (28) and (29):

(28) $${\left\|\mathrm{BM}\right\|}_{F}={\left(\sum_{i=1}^{n_{m}}\sum_{j=1}^{r}{\left|BM_{i,j}\right|}^{2}\right)}^{\frac{1}{2}}$$

(29) $${\left\|\mathrm{BD}\right\|}_{F}={\left(\sum_{i=1}^{n_{d}}\sum_{j=1}^{r}{\left|BD_{i,j}\right|}^{2}\right)}^{\frac{1}{2}}$$

Based on the above description, we have summarized the process of SCMC: the association probability matrix $A_{W}$ is used to construct the initial component matrices $M^{0}\in R^{n_{m}*r}$ and $D^{0}\in R^{n_{d}*r}$. According to formula (16), the initial probability matrix $P^{0}$ is calculated and the loss function is constructed, as shown in formula (18). Combined with the two weight matrices derived by the ANS algorithm, the loss function formula (18) is rewritten as formula (21) based on formulas (19) and (20); Using the loss function obtained from formula (21), calculate the partial derivatives of M and D based on formulas (22) and (23), and update the matrices M and D based on formulas (24) and (25). Repeat the above steps until $\max\left(∆M,∆D\right)<10^{-5}$; Finally, according to formula (17), calculate the correlation prediction matrix $P^{n+1}$ using $M^{n+1}$ and $D^{n+1}$.

## 3 RESULT

### 3.1 Assessment indicators

LOOCV and 5‐fold CV are used to evaluate the performance of our method and other state‐of‐the‐art microbe–disease prediction methods. In LOOCV, each known association between microorganisms and diseases is selected as a test sample, while other known associations are training samples. In 5‐fold CV, known associations are considered positive samples, and unobserved associations are considered negative samples. All positive samples were randomly divided into five groups, with four groups placed in the training set and the rest used for testing. In each CV, we randomly select negative samples with the same number of positive samples as the four groups for training, and the remaining negative samples are used for testing.

By sorting the samples using our method's scores and different thresholds, the ROC curve can be plotted and the area under the ROC curve (AUC) can be obtained.

#### 3.1.1 Optimal parameter selection

**FIGURE 3:**
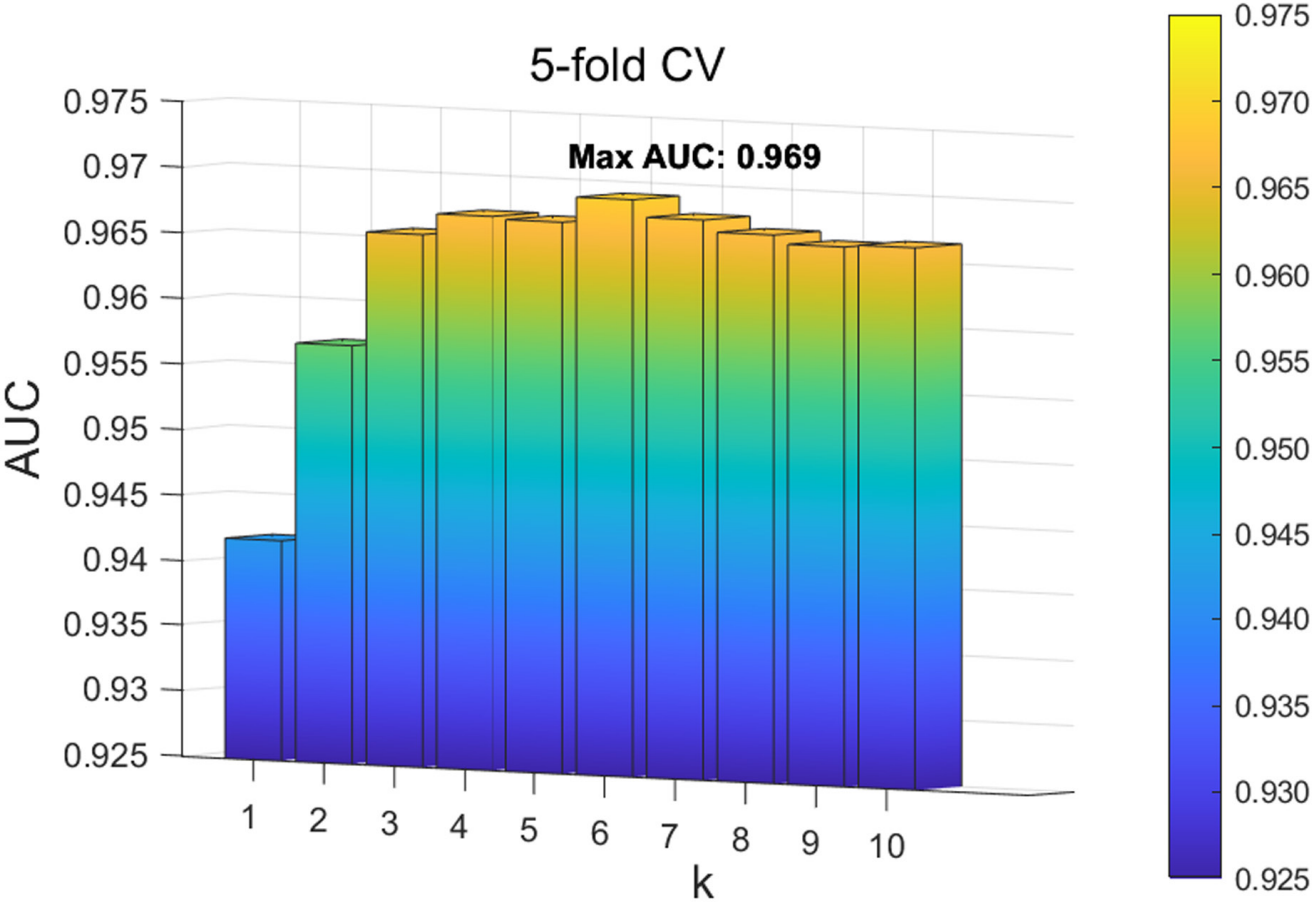
Effect of different K in WKNKN under 5‐fold CV.

**FIGURE 4:**
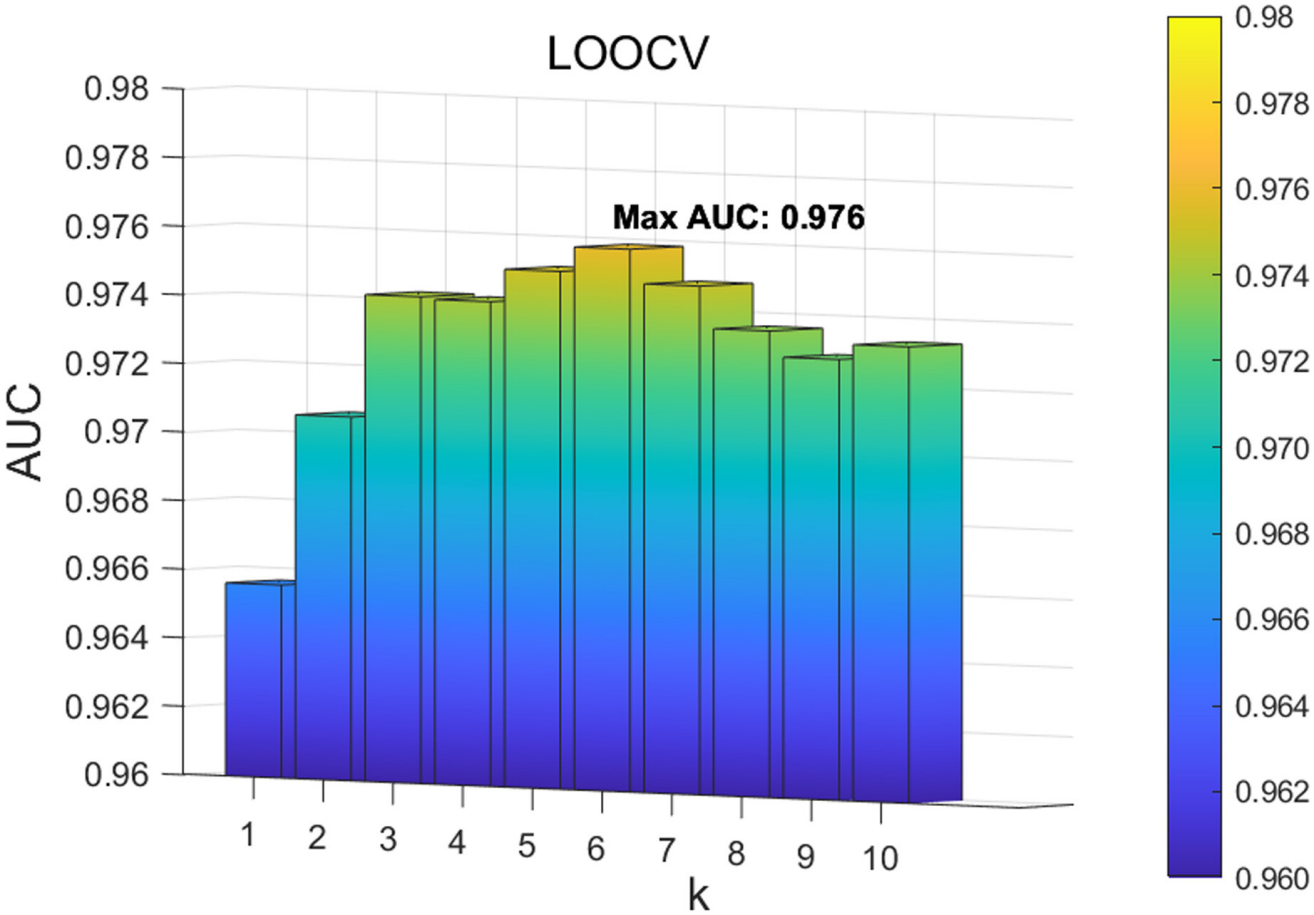
Effect of different K in WKNKN under LOOCV.

To evaluate the impact of the parameters in ANS‐SCMC, we analyse the impact of the parameters K of the WKNKN function to adjust the extraction of microbial features. We set the parameter K within the range $\left[1,10\right]$ with the step size of 1. The experimental results demonstrate that when the parameter K is set to 6, ANS‐SCMC achieves the best performance in both five‐fold CV and LOOCV, as shown in Figures 3 and 4. Therefore, we ultimately set the parameter K in WKNKN to 6.

**FIGURE 5:**
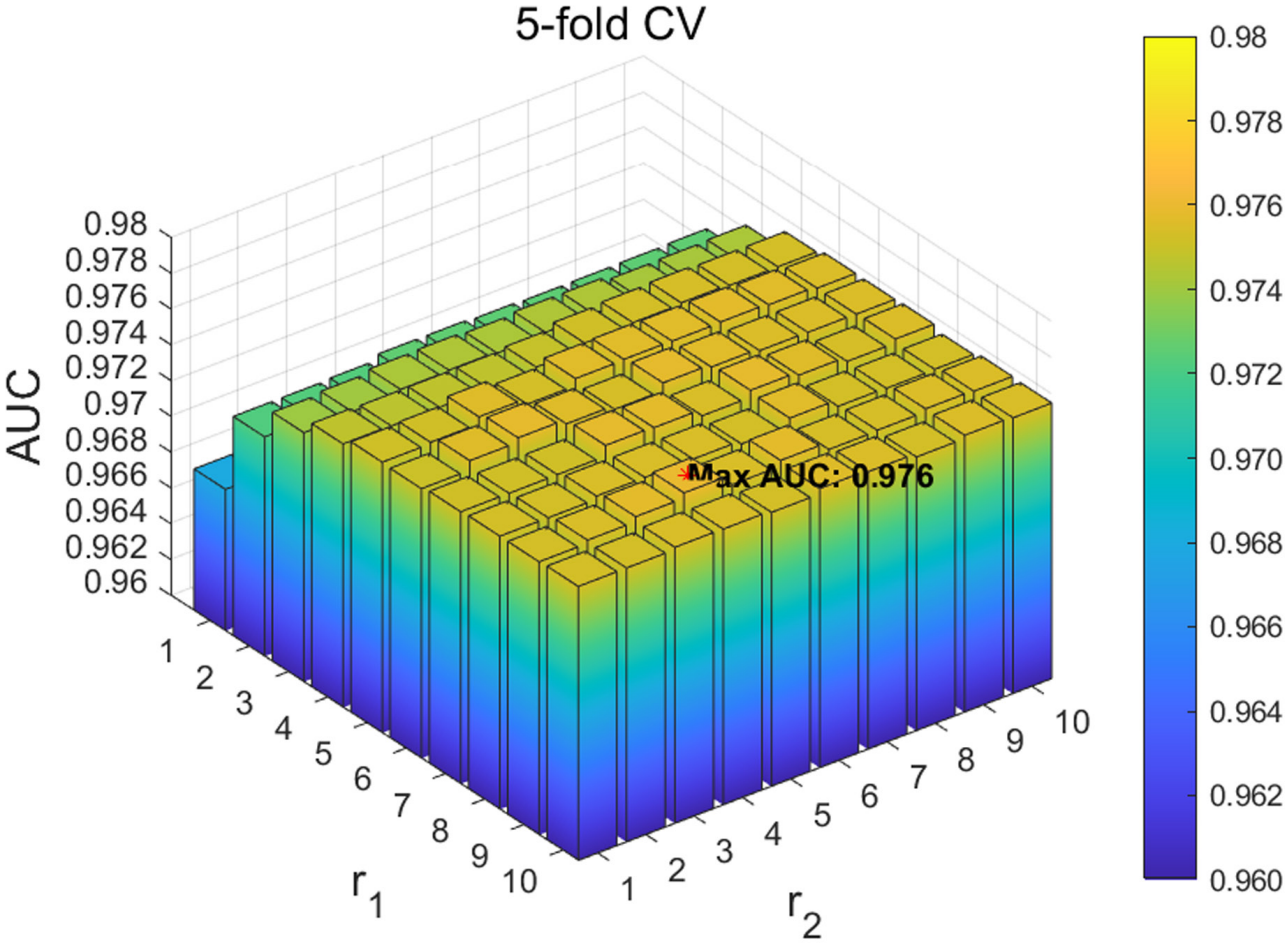
Effect of different $r_{1}$ and $r_{2}$ in ANS under 5‐fold CV.

**FIGURE 6:**
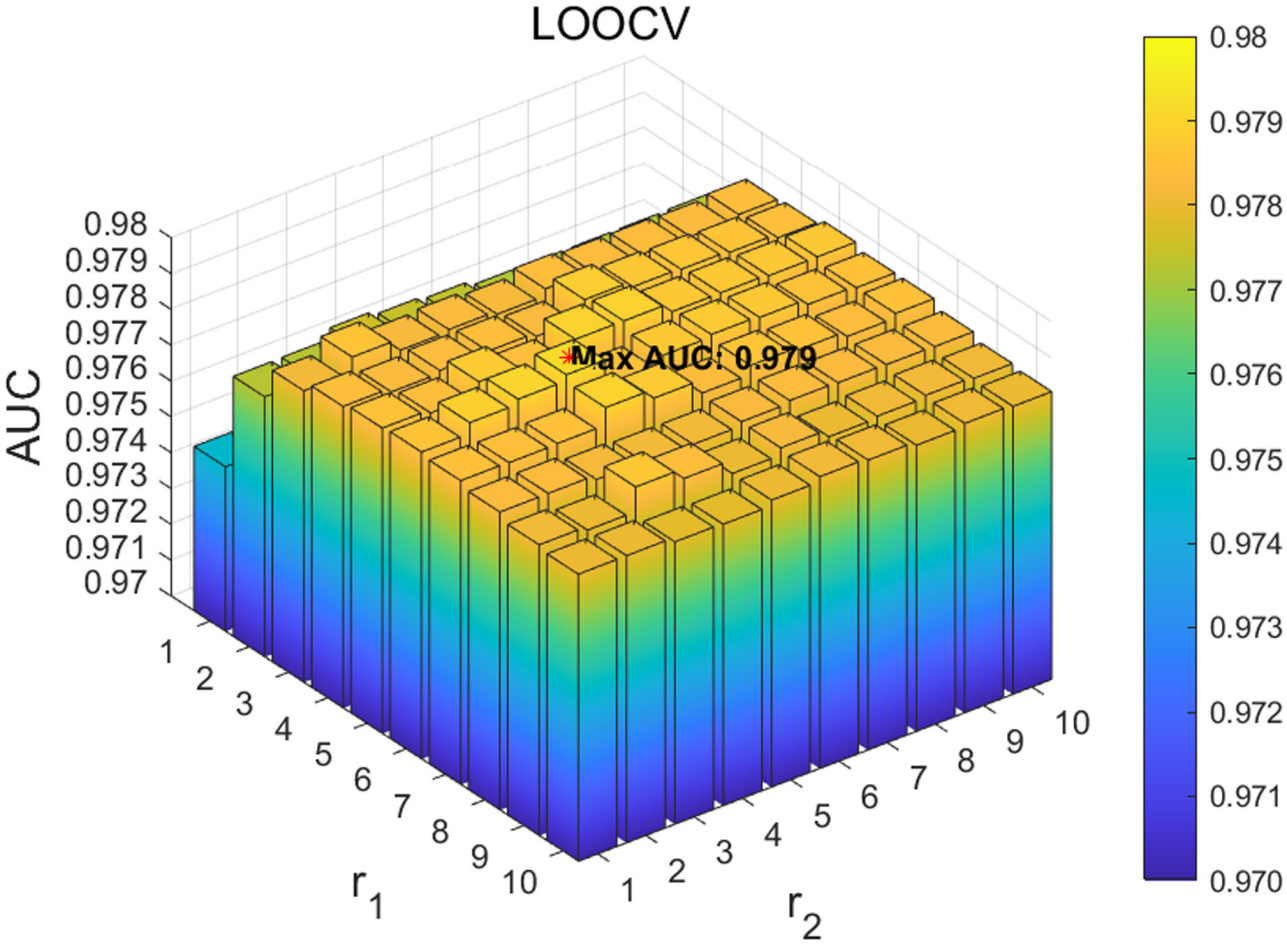
Effect of different $r_{1}$ and $r_{2}$ in ANS under LOOCV.

Next, we analysed the hyperparameters $r_{1}$ and $r_{2}$ in the adaptive similar matrix method ANS. Specifically, we focused on the two parameters $r_{1}$ and $r_{2}$ in the ANS method for obtaining microbial and disease weight matrices, with their ranges set to $\left[1,10\right]$ and step sizes set to 1. The experimental results show that when the parameters $r_{1}$ and $r_{2}$ are set to 9 and 4, respectively, ANS‐SCMC achieves the best performance in 5‐fold CV, as shown in Figure 5. When the parameters $r_{1}$ and $r_{2}$ are set to 6 and 4, ANS‐SCMC achieved the best performance in LOOCV, as shown in Figure 6. Based on the above results, we noticed that the prediction accuracy of the model in LOOCV gradually stabilized within the parameter $r_{1}\in \left[6,9\right]$ and reached saturation within the range. Based on observations of the data, we ultimately set the parameters $r_{1}$ and $r_{2}$ in ANS to 7 and 4, respectively.

**FIGURE 7:**
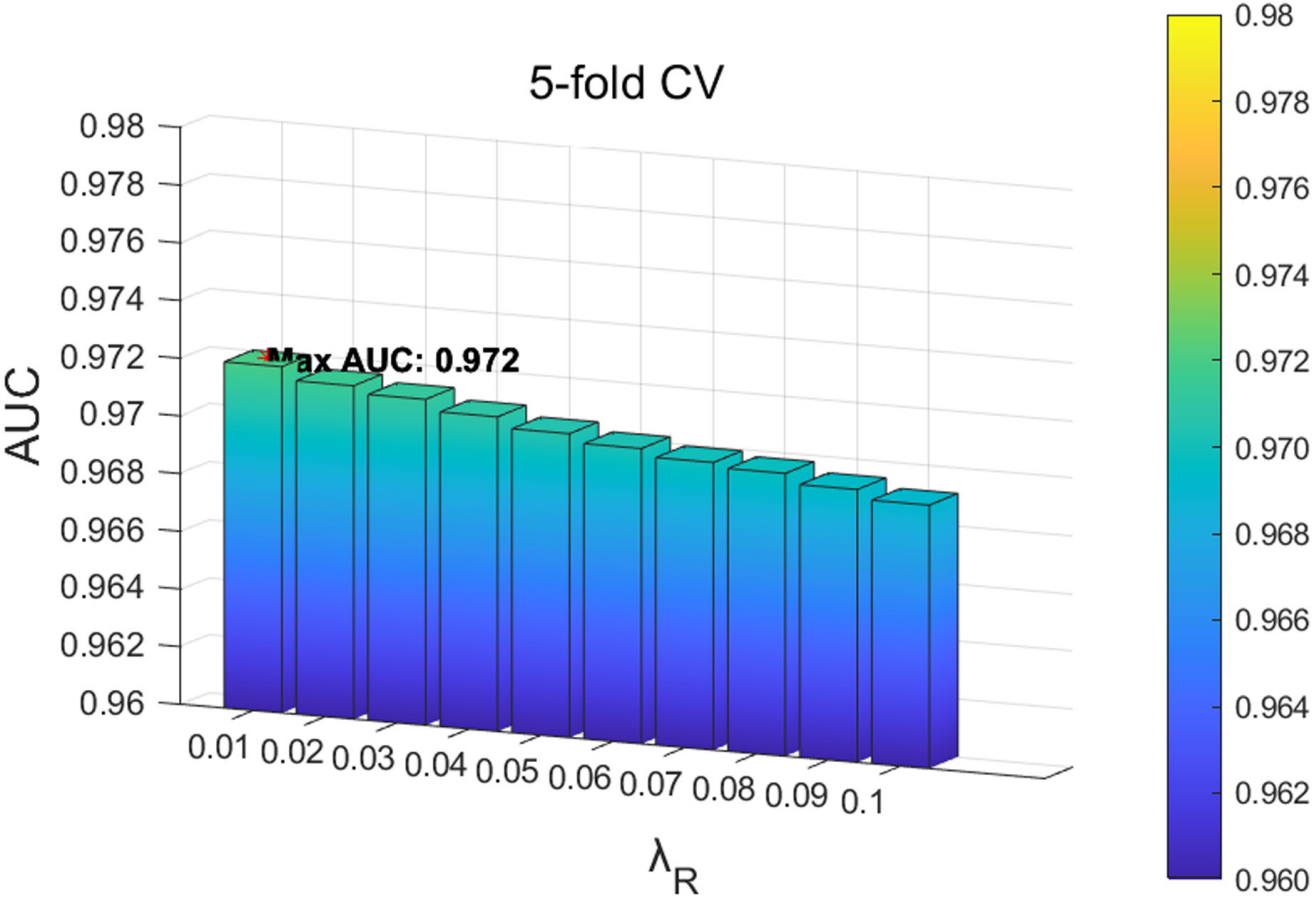
Effect of different $\lambda_{R}$ in SCMC under 5‐fold CV.

**FIGURE 8:**
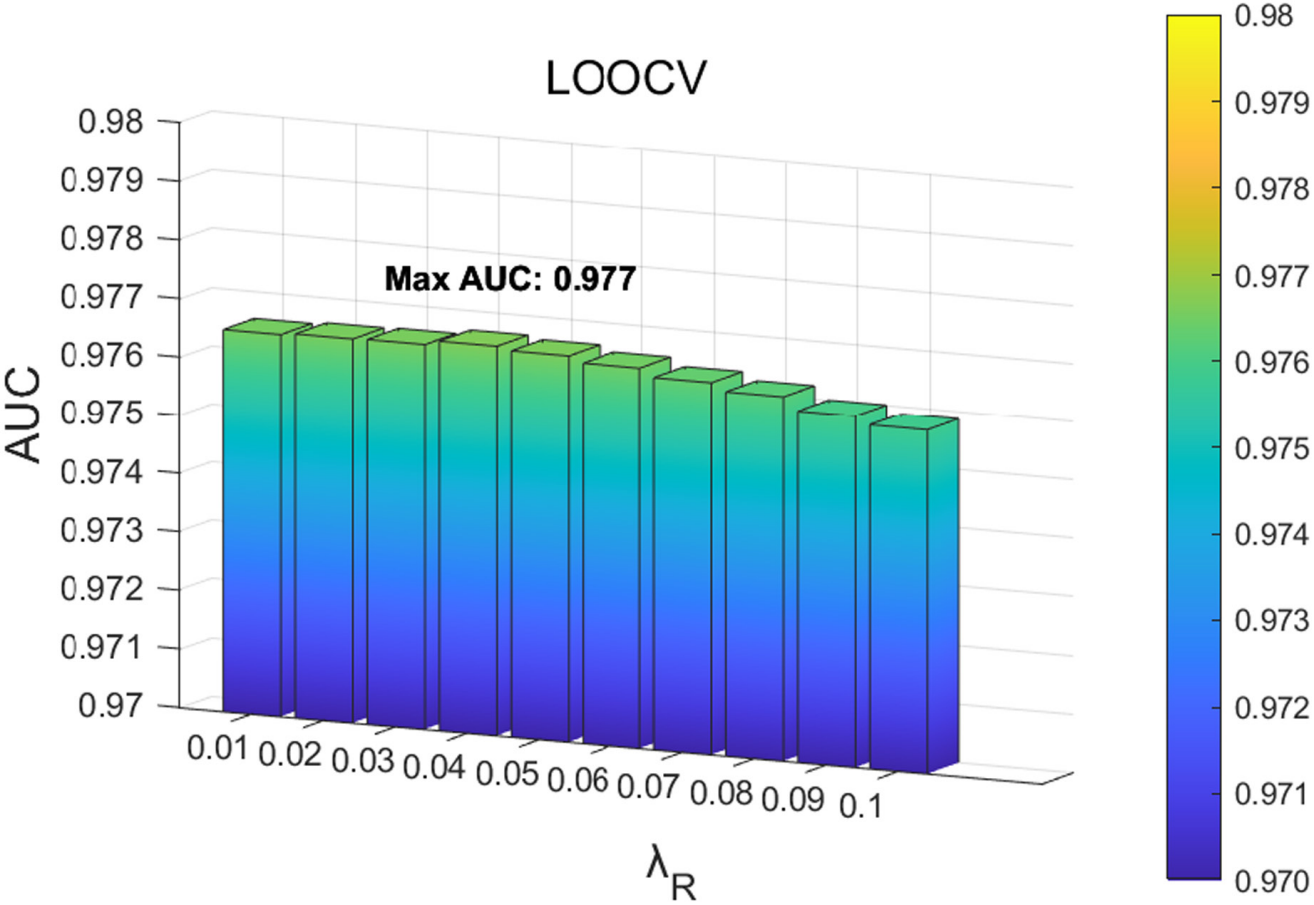
Effect of different $\lambda_{R}$ in SCMC under LOOCV.

**FIGURE 9:**
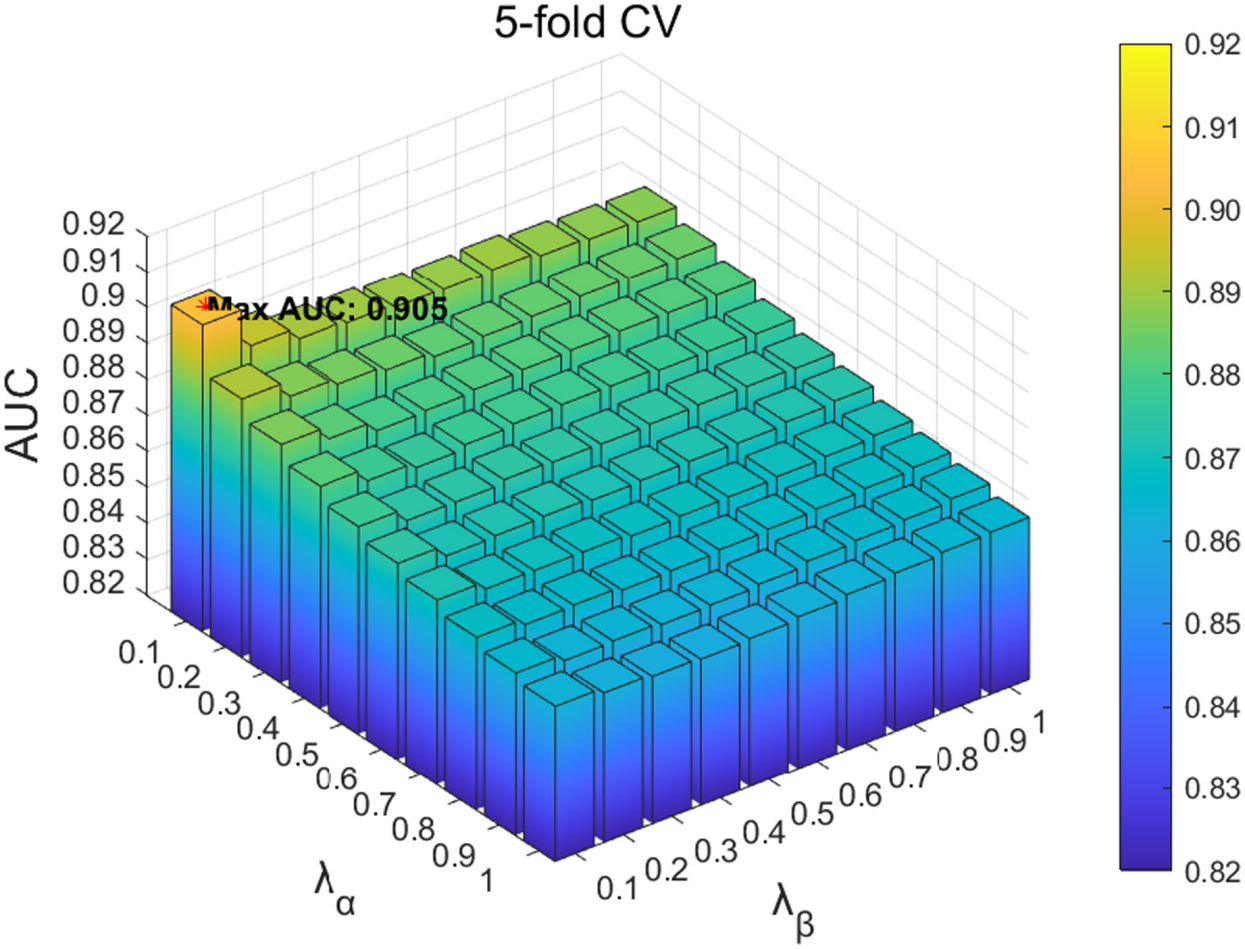
Effect of different $\lambda_{\alpha }$ and $\lambda_{\beta }$ in SCMC under 5‐fold CV.

**FIGURE 10:**
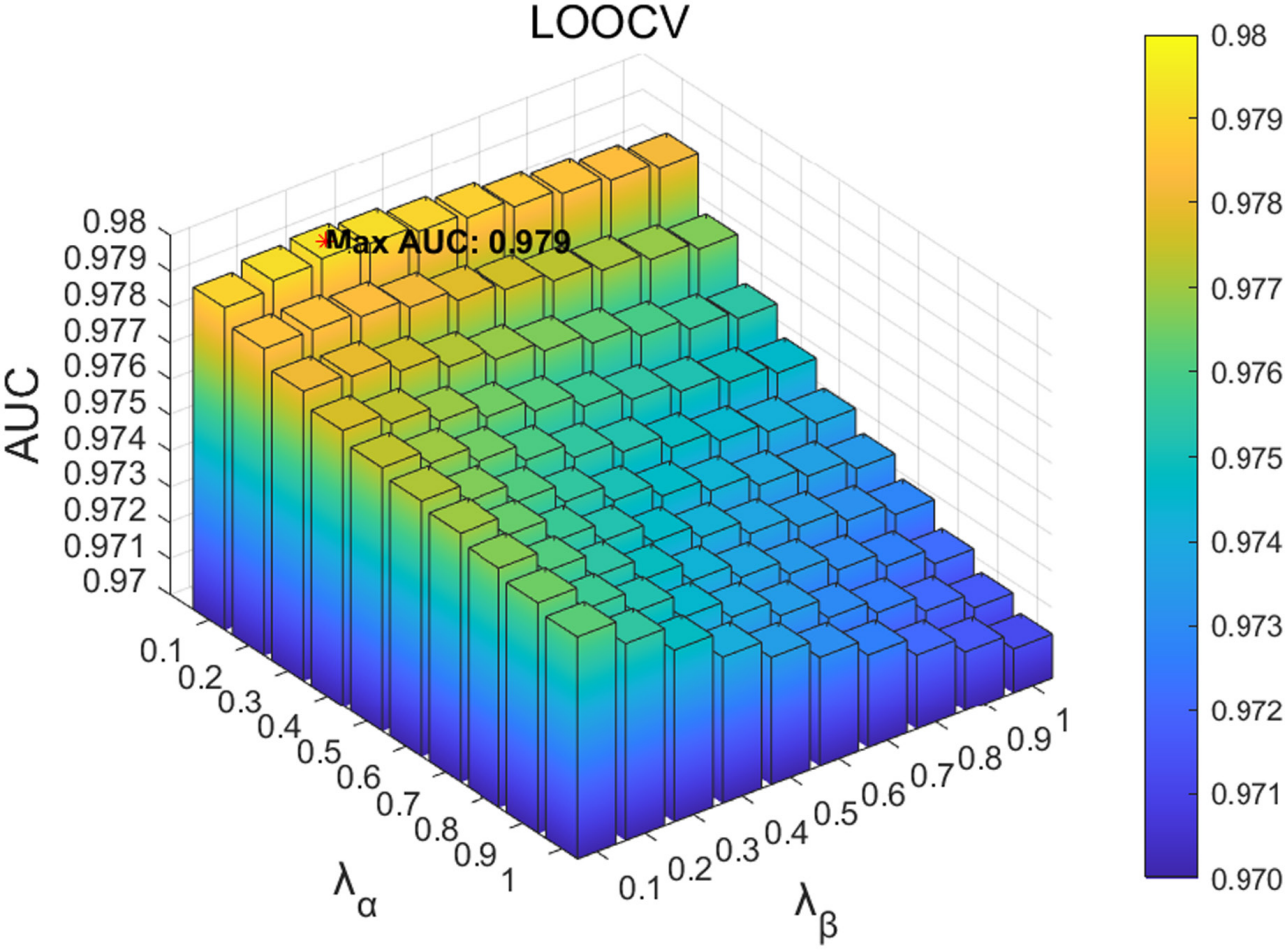
Effect of different $\lambda_{\alpha }$ and $\lambda_{\beta }$ in SCMC under LOOCV.

In the SCMC parameter tuning experiment, we first analysed the influence of parameter $\lambda_{R}$ in the loss function on the function. The value range of parameter $\lambda_{R}$ was located at $\left[0.01,0.1\right]$, and adjusted with a step size of 0.01. According to the experimental results, when the value of $\lambda_{R}$ is 0.01, the model performs best in 5‐fold CV, as shown in Figure 7; When the value of parameter $\lambda_{R}$ is 0.04, it performs best in LOOCV, as shown in Figure 8. Based on the above results, considering that the prediction accuracy of the model in LOOCV gradually stabilizes and reaches saturations at parameter $\lambda_{R}\in \left[0.01,0.04\right]$, we ultimately set the parameter $\lambda_{R}$ in SCMC to 0.01. Next, we investigated the influence of parameters $\lambda_{\alpha }$ and $\lambda_{\beta }$ on the loss function, with a range of $\left[0.1,1\right]$ and a step size of 0.1. When the parameters $\lambda_{\alpha }$ and $\lambda_{\beta }$ are 0.1 and 0.1 respectively, the 5‐fold CV achieved the best performance, as shown in Figure 9; When parameters $\lambda_{\alpha }$ and $\lambda_{\beta }$ are 0.1 and 0.3, respectively, LOOCV achieved the best performance, as shown in Figure 10. Based on these findings and the fact that the prediction accuracy of the model in LOOCV gradually stabilizes and reaches saturation at parameter $\lambda_{\beta }\in \left[0.1,0.\;3\right]$, we ultimately set the parameters $\lambda_{\alpha }$ and $\lambda_{\beta }$ in SCMC both to 0.1. Therefore, we set the parameter $\lambda_{R}$ in SCMC to 0.01, and the parameters $\lambda_{\alpha }$ and $\lambda_{\beta }$ to 0.1 and 0.1, respectively.

### 3.2 Algorithm comparison

**FIGURE 11:**
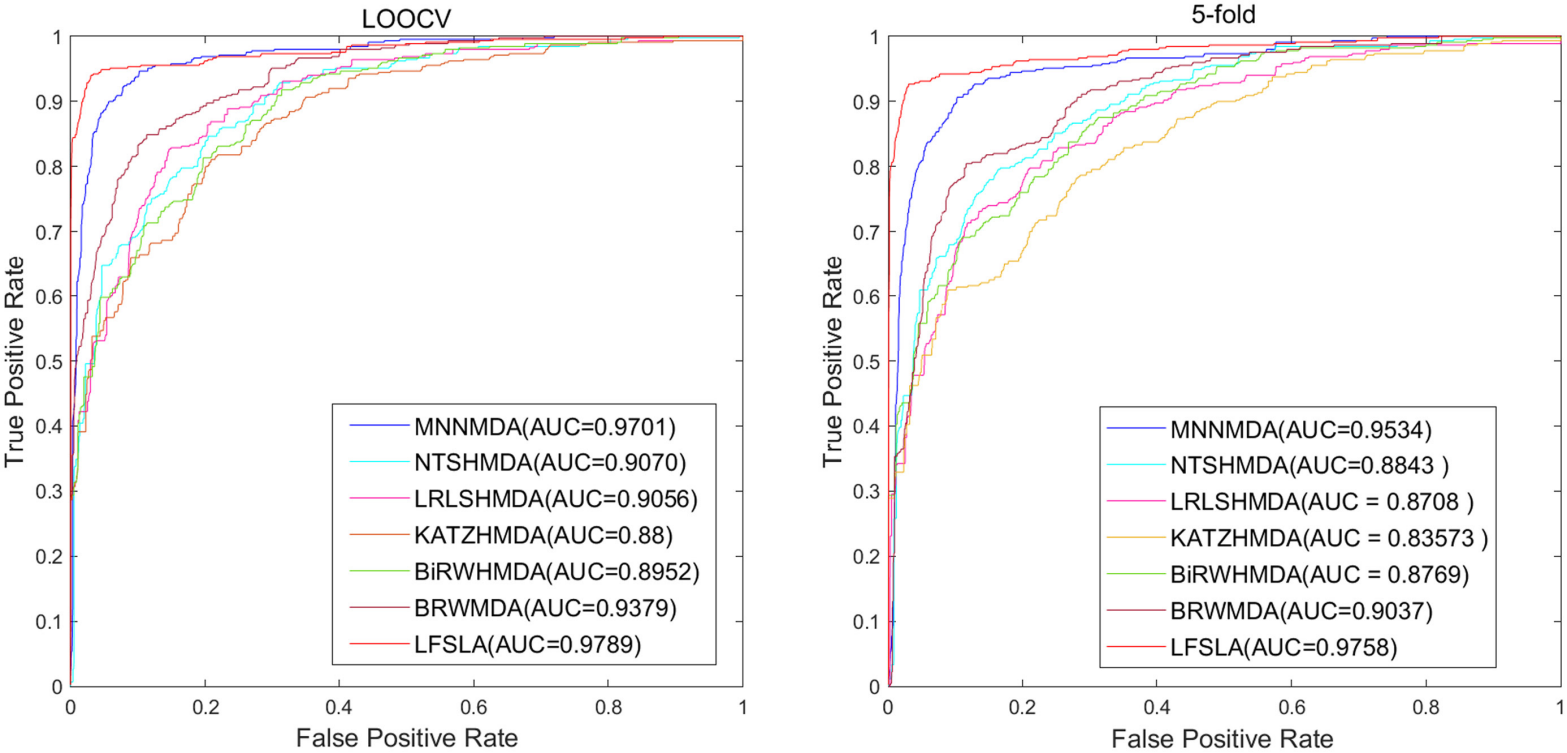
Results of ANS‐SCMC comparison experiments.

We evaluated the MDA prediction performance of the proposed ANS‐SCMC method on the HMDMD dataset, using LOOCV and five‐fold CV, and compared it with six other MDA identification methods. These methods are MNNMDA,^36^ NTSHMDA,^37^ LRLSHMDA,^38^ KATZHMDA,^39^ BiRWHMDA^40^ and BRWMDA.^41^ The experimental results are shown in Figure 10. We found that ANS‐SCMC exhibits excellent performance on HMDAD, with the highest AUC values of 0.9789 on LOOCV and 0.9758 on five‐fold CV, respectively. Overall, compared with the other six methods, ANS‐SCMC has excellent predictive performance (Figure 11).

### 3.3 Case studies

In our study, we generated a training set from a known microbial disease association dataset to evaluate the effectiveness of our proposed ANS‐SCMC method in predicting unknown microbial disease associations. Through this method, we generated an association prediction score for each unknown microbial disease pair and ranked these scores in descending order. Next, our goal is to find new microbes to treat type 1 diabetes and Crohn's disease (CD).

#### 3.3.1 Type 1 diabetes

Type 1 diabetes is a chronic autoimmune disease that causes the pancreas to produce very little or no insulin.^42^ Insulin is a hormone that helps blood sugar enter body cells and convert it into energy. Lack of insulin can lead to blood sugar not being absorbed by cells, but accumulating in the bloodstream, resulting in high blood sugar and related symptoms and complications.^43^ Type 1 diabetes was previously known as insulin dependent diabetes or juvenile diabetes, but it can actually occur at any age, although it only accounts for 5%–10% of all diabetes cases. There are currently no known preventive measures.^44^

In our study, the ANS‐SCMC method was used to explore new microbes related to type 1 diabetes. In the HMDAD database, we predicted the first 10 microbes that may be related to type 1 diabetes. Among these 10 potential related microbes, 8 have been validated in the literature. In addition, our study also suggests that Whipple barrier organism and oxalobacter may be associated with type 1 diabetes. As shown in Table 1.

**TABLE 1:** Predicted top 10 microbes associated with type 1 diabetes by ANS‐SCMC.

Disease Name	Name of microbes	Corresponding literature
Type 1 diabetes	Bacillariophyta (phylum of warts and microfungi)	29988362
Type 1 diabetes	*Clostridium difficile* (bacterium causing gut infection)	34040023
Type 1 diabetes	*Clostridium nucleatum*	25294115
Type 1 diabetes	*Pseudomonas* genus (bacteria)	37279395
Type 1 diabetes	Desulfurization bacteria	33106354
Type 1 diabetes	Whipple's Body of Nutritional Barriers	Unconfirmed
Type 1 diabetes	Prevotella	36562032
Type 1 diabetes	Bacillus oxalic acid bacteria	Unconfirmed
Type 1 diabetes	Enterococci	36603588
Type 1 diabetes	Propionibacterium acnes (the bacterium that causes acne)	18500429

#### 3.3.2 Crohn's disease (CD)

Crohn's disease (CD) is an inflammatory bowel disease that may affect any part of the digestive tract. Its symptoms include abdominal pain, diarrhoea, fever, bloating, and weight loss.^45^ The exact cause of Crohn's disease is not yet clear, but it may be related to genetics, immune response, environmental factors, and gut microbiota. Although there is currently no cure for Crohn's disease, symptoms can be managed through medication, surgery, or lifestyle adjustments. Crohn's disease may cause various complications, such as intestinal obstruction, intestinal fistula, intestinal perforation, malnutrition, anaemia, and other related inflammations.^46^

In this study, we applied the ANS‐SCMC method to identify microbes that may be associated with Crohn's disease. In the MDA database, we predicted the top 10 microbes that may be associated with Crohn's disease, all of which were validated by the database or existing literature. As shown in Table 2.

**TABLE 2:** Predicted top 10 microbes associated with Crohn's disease by ANS‐SCMC.

Disease Name	Name of microbes	Corresponding literature
Crohn's disease	*Clostridium difficile* (bacterium causing gut infection)	31562236
Crohn's disease	*Helicobacter pylori*	35951774
Crohn's disease	Bifidobacterium (bifidus)	37240476
Crohn's disease	Mycobacterium fragileis (genus of sac fungus, a plant pathogen)	34168621
Crohn's disease	Prevotella	32730134
Crohn's disease	*Clostridium difficile* (bacterium causing gut infection)	26951181
Crohn's disease	Tricholoma (family of fungi)	35860271
Crohn's disease	*Escherichia coli* (*E*. *coli*)	37170220
Crohn's disease	Ascomycota, the phylum of fungi	31170412
Crohn's disease	*Enterococcus faecalis* (genus *Enterococcus*)	20E722058

## 4 DISCUSSION AND CONCLUSION

This study proposes the ANS‐SCMC method, aiming to discover new connections between microbes and complex human diseases. The ANS‐SCMC method first calculates the Gaussian similarity between microbes and diseases. It then utilizes the WKNKN algorithm to extract features and compute pairwise associations between microbes and diseases. Subsequently, it constructs adaptive neighbourhood similarity for both microbes and diseases individually. Finally, the SCMC method is employed to decompose the preprocessed loss function matrix into two low‐rank matrices. The missing values in the original matrix are computed by iteratively updating the matrices using gradient descent. This allowed the two sub matrices to continuously approach the original matrix, resulting in the final score for disease microbial association prediction. We compared ANS‐SCMC with six currently advanced MDA identification models in the HMDAD database and achieved the highest AUC values in both five‐fold CV and LOOCV. In addition, we used ANS‐SCMC in case studies to predict microbes related to type 1 diabetes and Crohn's disease. We compared the predicted top 10 microbes with the actual results, and the prediction accuracy in the verified microbe–disease association was 80% and 100% respectively. The results show that Whipple barrier organism and oxalobacter may be closely related to type 1 diabetes, and this discovery needs further biological experiments to verify.

In the future, we plan to design more accurate negative MDA screening methods by combining the biological characteristics of microbes, diseases, and MDA networks. We will also develop new deep learning models to improve the performance of MDA classification based on reliable negative MDA samples. We hope that the proposed ANS‐SCMC method can help identify disease‐related microbes and provide clues for therapeutic research.

## AUTHOR CONTRIBUTIONS

**Haoran Wen:** Conceptualization (equal); data curation (equal); formal analysis (equal); funding acquisition (equal); investigation (equal); methodology (equal); project administration (equal); validation (equal); visualization (equal); writing – original draft (equal); writing – review and editing (equal). **Xue Zhong:** Conceptualization (equal); data curation (equal); formal analysis (equal); investigation (equal); visualization (equal); writing – original draft (equal); writing – review and editing (equal). **Lieqing Lin:** Conceptualization (equal); data curation (equal); formal analysis (supporting); methodology (supporting); validation (supporting); visualization (supporting); writing – review and editing (equal). **Langcheng Chen:** Conceptualization (equal); data curation (equal); formal analysis (supporting); methodology (supporting); validation (supporting); writing – review and editing (equal).

## CONFLICT OF INTEREST STATEMENT

The authors declare that the research was conducted in the absence of any commercial or financial relationships that could be construed as a potential conflict of interest.

## Data Availability

Code and datasets are available at: https://github.com/kevin9972/ANS‐SCMC.
